# The differential diagnostic value of selected cardiovascular biomarkers in Takotsubo syndrome

**DOI:** 10.1007/s00392-021-01956-2

**Published:** 2021-11-02

**Authors:** Albert Topf, Moritz Mirna, Vera Paar, Lukas J. Motloch, Janine Grueninger, Christiane Dienhart, Paul C. Schulze, Mathias C. Brandt, Robert Larbig, Uta C. Hoppe, Daniel Kretzschmar, Michael Lichtenauer

**Affiliations:** 1 Clinic for Internal Medicine II, Department of Internal Medicine II, Paracelsus Medical University, University Hospital Salzburg, Paracelsus University Salzburg, Müllner Hauptstraße 48, 5020 Salzburg, Austria; 2 Department of Internal Medicine I, Paracelsus Medical University, 5020 Salzburg, Austria; 3 Division of Cardiology, Department of Internal Medicine I, University Hospital Jena, 07743 Jena, Germany; 4 Devision of Cardiology, Hospital Maria Hilf Moenchengladbach, 41063 Möenchengladbach, Germany

**Keywords:** Takotsubo cardiomyopathy, Acute coronary syndrome, Biomarkers, Differential diagnosis

## Abstract

**Introduction:**

Takotsubo syndrome (TTS) is clinically indistinguishable from an acute coronary syndrome (ACS). In the absence of valid markers for differential diagnosis, coronary angiography has been indispensable.

**Methods:**

In our study, we evaluated the serum levels of sST-2, GDF-15, suPAR and H-FABP in 92 patients with the suspicion of TTS (51 TTS and 41 ACS patients) and 40 gender matched controls (no coronary artery disease or signs of heart failure) at baseline.

**Results:**

H-FABP was significantly higher in ACS patients compared to TTS patients. Even in in propensity score matching for left ventricular ejection fraction, sex and cardiovascular risk factors, differences in the plasma levels of H-FABP in the matched cohort of TTS vs ACS remained statistically significant. Whereas, sST-2 was significantly elevated in TTS patients. H-FABP was superior for prediction of an ACS with even higher accuracy than hs troponin in differential diagnosis (AUC 0.797, *p* ≤ 0.0001); the optimal cut off for discrimination towards a TTS was calculated as 2.93 ng/ml (sensitivity 70.0%, specificity 82.4%, PPV 75.7%, NPV 77.4%). sST-2 seemed most appropriate for identification of a TTS (AUC 0.653, *p* = 0.012). The optimal cut off for differential diagnosis was 11018.06 pg/ml (sensitivity 82.0%, specificity 51.2%, PPV 69.4%, NPV 71.9 %).

**Conclusion:**

H-FABP and sST-2 are the most promising markers with better accuracy than preexisting biomarkers in differential diagnosis in our study and therefore, could be crucial for the guidance of treatment in patients with high bleeding risk, advanced renal failure or multimorbidity.

**Graphical abstract:**

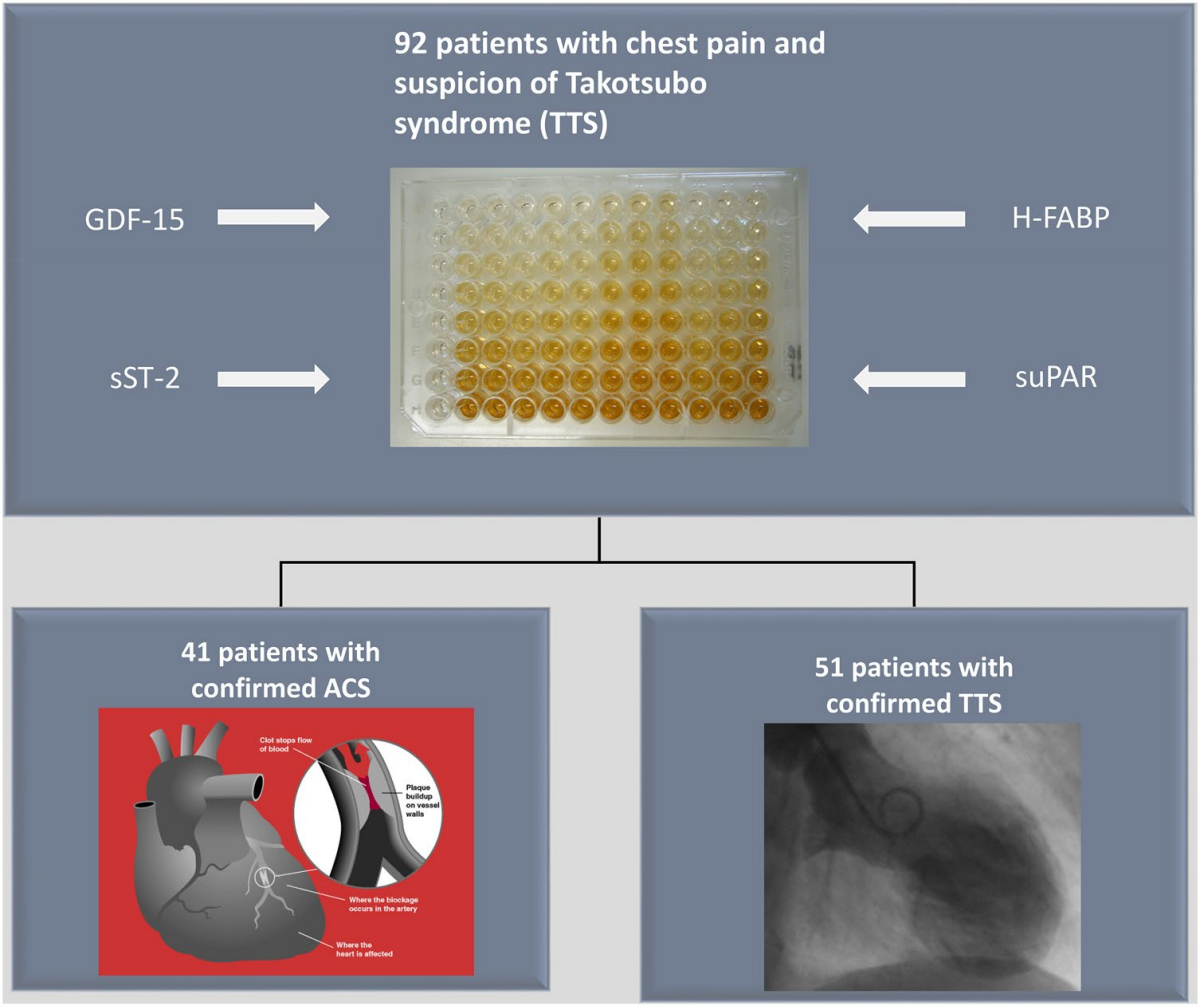

**Supplementary Information:**

The online version contains supplementary material available at 10.1007/s00392-021-01956-2.

## Introduction

Takotsubo syndrome (TTS) is an acute heart failure condition characterized by acute left ventricular dysfunction with symptoms similar to an acute myocardial infarction, but in absence of significant coronary stenosis [1]. Three percent of all suspected acute coronary syndromes (ACS) are caused by TTS, with higher propensity in females (incidence of up to 7.5% in the female population) [2]. The syndrome is often triggered by emotional and physical stress factors and comprises reversible wall motion abnormalities involving apical, midventricular or basal segments of the left ventricle [3]. 

The pathophysiological mechanism of TTS has not been clearly elucidated. It has been suggested that in TTS, the myocardium responds to excessive epinephrine release with myocardial stunning [4].

Patients with TTS usually have a good prognosis; in 96% of the cases, an almost full recovery with resolution of wall motion abnormalities can be observed within a few days [5]. Nevertheless, along with the high percentage of morbidity, the acute phase can also be life-threatening (1–2% mortality). In the acute phase, there is a 20% risk of congestive heart failure. Furthermore, life-threatening ventricular arrhythmias occur in 8.6% of TTS patients and left ventricular wall rupture, thrombosis and even cardiogenic shock have been reported in the acute phase [6].

A key issue in clinical practice is that TTS is especially difficult to distinguish from ACS. Several biomarkers are available that may contribute to differentiation between TTS and ACS. Although, there are scoring systems which attempt to increase prediction of TTS, coronary angiography currently remains necessary for differentiating between these two syndromes [7].

In this study, we investigated a selected spectrum of novel cardiovascular biomarkers for their differential diagnostic value in TTS. We chose markers already well studied in other cardiovascular diseases, including heart failure and acute coronary syndrome [8, 9].

One of the markers which is best studied and most frequently applied in clinical practice, is soluble suppression of tumorigenicity (sST-2). sST-2 is a member of the interleukin-1 (IL-1) receptor family, which is known to act as a membrane-bound receptor (ST2L), but also as a secreted protein (soluble ST-2; sST-2) [10]. The functional ligand for the ST2L receptor is Interleukin-33 (IL-33). IL-33 secretion is triggered by local tissue inflammation and also by necrotic cell death as a danger signal [11]. Expressed by cardiomyocytes and cardiac fibroblasts, an excess of sST-2 leads to binding and subsequent reduced bioavailability of circulating cardioprotective ligand IL-33, which reduces apoptosis and improves myocardial function. Furthermore, it has been implicated as a marker of cardiac mechanical strain [12].

Growth-differentiation factor-15 (GDF-15) is a member of the transforming growth factor β-family and has also been described as a stress-responsive biomarker of cardiac and vascular disease. GDF-15 expression is up-regulated in the presence of oxidative stress and inflammation. Its prognostic value in the setting of acute non-ST-elevation myocardial infarction (NSTEMI) has been reported [13].

Soluble urokinase plasminogen activator receptor (suPAR) is a proinflammatory marker, which is associated with systemic inflammatory response syndrome, malignancies, and cardiovascular disease. Furthermore, suPAR is expressed in a variety of cells, which play a critical role in all stages of atherogenesis—from the initiation of fatty streaks to progression of atherosclerosis and plaque destabilization. Plasma levels of suPAR correlate with atherosclerosis and with individual’s risk for cardiovascular disease, type 2 diabetes mellitus, cancer, as well as mortality [14].

Heart-type fatty acid binding protein (H-FABP) is a low molecular weight protein which is expressed in cardiomyocytes. Similar to troponin, H-FABP is released in the presence of myocardial damage, such as ischemia, which is why it is considered an early indicator for ischemic heart damage. Elevated H-FABP levels at hospital admission are predictors of a lethal outcome, as well as non-fatal cardiac adverse events, even in absence of troponin elevations [15].

Among the investigated biomarkers, quick tests for H-FABP and sST-2 are available. Quick tests for H-FABP, with results within 15 min, have provided diagnostic value in primary care for detection of ACS. Authors suggested that H-FABP quick tests can provide more certainty in diagnosing ACS [16].

The measurement of sST2 by the ASPECT-PLUS ST2 test provides results within 35 min with similar accuracy to the standard PRESAGE ST2 assay [17].

The aim of this study is to investigate the differential diagnostic value of these novel biomarkers to distinguish TTS from ACS.

## Methods

### Patients and controls

The study was approved by the local ethic committee (415-E/2230/10-2018) and was performed in accordance with the Declaration of Helsinki and Good Clinical Practice. All patients provided written informed consent prior to enrollment.

In this study, we recruited 92 consecutive patients hospitalized for chest pain and the suspicion of TTS in 2 study centers in Salzburg and Jena. 51 patients with TTS were enrolled, if they fulfilled the Mayo Clinic Diagnostic Criteria for TTS [18]. In total, 41 patients with an ACS were included. ACS was diagnosed and treated in accordance with the European Society of Cardiology criteria [19]. We also recruited 40 gender-matched healthy subjects, without coronary artery disease or echocardiographic signs of heart failure, for our control group.

Serum samples were collected within 24 h after the onset of symptoms. Data on clinical presentation, precipitating factors, cardiovascular risk factors, medications, and demographics were obtained as well.

### Blood samples

Blood samples were collected from a cubital vein using a sterile technique under controlled venous stasis. The collection tubes were centrifuged within 20 min after blood collection and the obtained plasma samples were frozen at − 80 °C until further analysis was performed. Routine blood analysis, according to our clinical standards, was also performed at the time of initial study sample collection.

### Transthoracic echocardiography

Transthoracic echocardiography at baseline (Philips iE 33 ultrasound system) was used to assess left ventricular ejection fraction (LVEF). Standard echocardiographic views, including parasternal long axis view, parasternal short axis view and apical four-chamber view, were acquired as previously published [20]

### Biomarker analysis

Serum biomarker analysis was performed at baseline as well as at follow-up at 1 month. Levels of sST-2, GDF-15, suPAR, and H-FABP were measured using commercially available enzyme-linked immunosorbent assay (ELISA) kits (DuoSet ELISA, DY523B, R&D Systems, Minneapolis, MN, USA). ELISA assays were performed in accordance with instructions supplied by the manufacturer. In short, serum samples and standard proteins were added to the multiwell plate coated with the respective capture antibody and incubated for 2 h. Plates were then washed using washing buffer (Tween 20, Sigma Aldrich, USA and phosphate buffered saline solution). In the next step, a biotin-labelled antibody was added to each well and incubated for an additional 2 h. After incubation, the ELISA plates were washed and a streptavidin–horseradish-peroxidase solution was added. After adding tetramethylbenzidine (TMB; Sigma-Aldrich, USA), a color reaction was achieved. Optical density was measured at 450 nm on an ELISA platereader (iMark Microplate Absorbance Reader, Bio-Rad Laboratories, Austria).

### Statistical analysis

Statistical analysis was performed using SPSS (22.0, SPSS Inc., USA) and R (version 4.0.2., R Core Team (2013), R Foundation for Statistical Computing, Vienna, Austria; http://www.R-project.org/) with the packages ‘ggplot2’, ‘glmnet’, ‘pastecs’, ‘Hmisc’, ‘ggm’, ‘QuantPsyc’., ‘Matching’, ‘MatchIt’, ‘optmatch’, ‘RItools’ and ‘Rcpp’. The Kolmogorov–Smirnov test was used to assess distribution of data in the study population. As most parameters and biomarker concentrations were not normally distributed, all values were given as median and interquartile range (IQR). Median values between groups were compared by Mann–Whitney *U* test or Kruskal–Wallis test with Dunn’s post hoc test. Correlation analysis was performed using Spearman’s rank-correlation coefficient. ROC analysis was performed and an optimal cut-off was calculated by means of the Youden Index. Areas under the curve (AUC) were compared as described by Hanley and McNeil [21]. Prior to propensity score matching, covariate imbalances were evaluated by calculating standardized/normalized differences and performing a Chi-square test. Propensity score matching was conducted using near neighbor with caliper matching with *ε* < 0.1 *σp*. A *p* < 0.05 was considered to be statistically significant.

## Results

### Baseline characteristics

Baseline characteristics of control patients and those suffering from TTS or ACS are shown in Table 1. TTS patients were non-significantly older than patients with ACS (*p* = 0.838). Female patients were almost similar distributed between the TTS (94.1%, *n* = 48) and ACS subgroup (92.5%, *n* = 37; *p* = 0.783). Left ventricular ejection fraction of patients with TTS was significantly lower than of patients with ACS (*p* < 0.001). The apical type of TTS was the most frequent (90.2%, *n* = 46), followed by the midventricular (7.8%, *n* = 4) and the basal type (2%, *n* = 1). In the ACS subgroup, LAD was the main culprit lesion (85.4%, *n* = 35), followed by RCX (9.8%, *n* = 4) and RCA (4.9%, *n* = 2). Pro-BNP levels were significantly higher in TTS patients than in ACS patients (*p* = 0.043), whereas hs-troponin levels were significantly higher in ACS compared to TTS (*p* < 0.001).

**Table 1:** Baseline characteristics patients suffering from TTS or ACS and controls, given as median and IQR

	TTS		ACS		*p* =	Control	
	Median	IQR	Median	IQR		Median	IQR
Age (years)	74.0	62.0–78.0	71.0	61–78	0.838	67.0	54.3–73.0
BMI (kg/m^2^)	24.7	21.8–29.2	28.7	25.0–33.2	0.004	26.6	23.2–31.0
EF (%)	40.0	35.0–46.0	52.6	40.8–61.0	< 0.001	67.0	62.3–73.0
Creatinine (µmol/l)	64.2	59.8–79.2	72.0	62.0–85.4	0.168	68.0	64.0–76.8
LDL (mg/dl)	90.0	75.0–122.0	126.5	102.1–151.8	0.012	125.8	108.3–172.1
CRP (mg/l)	0.4	0.2–0.9	4.8	0.0–9.7	0.039	3.4	2.7–5.4
HbA1c (%)	5.4	5.2–5.8	6.1	5.6–6.8	0.001	6.0	5.7–6.5
(hs) Troponin (pg/ml)	162.0	53.0–395.0	564.3	230.8–1951.0	< 0.001	5.4	3.7–9.4
Pro-BNP (pg/ml)	2866.0	664.6–4919.8	1059.0	636.9–2325.5	0.043	111.5	32.7–292.9
sST-2 (pg/ml)	24354.9	13071.5–47468.3	10302.3	8019.5–41559.8	0.012	4957.0	3631.2–5781.4
H-FABP (ng/ml)	1.1	0.6–2.3	5.3	2.2–20.7	< 0.001	0.0	0.0–0.0
suPAR (pg/ml)	3076.8	2350.3–4118.0	3656.6	2705.4–6649.8	0.032	2680.4	2267.9–3501.0
GDF-15 (pg/ml)	924.8	610.7–1529.3	1009.2	619.5–1663.8	0.522	573.8	423.5–708.3
Smoking	15/51 (29.4%)		16/41 (39.0%)			05/40 (12.5%)	
Hypertension	38/51 (74.5%)		36/41 (87.8%)			33/40 (82.5%)	
Sex (female)	48/51 (94.1%)		37/41 (92.5%)			38/40 (92.7%)	

*p* = significance between TTS and ACS patients

ACS patients showed significantly higher LDL (*p* = 0.012) and HbA1c levels (*p* = 0.001) than TTS patients. Regarding comorbidities, hypertension was significantly more often present in patients with an ACS compared to TTS patients (*p* = 0.030).

### Biomarkers

**Fig. 1:**
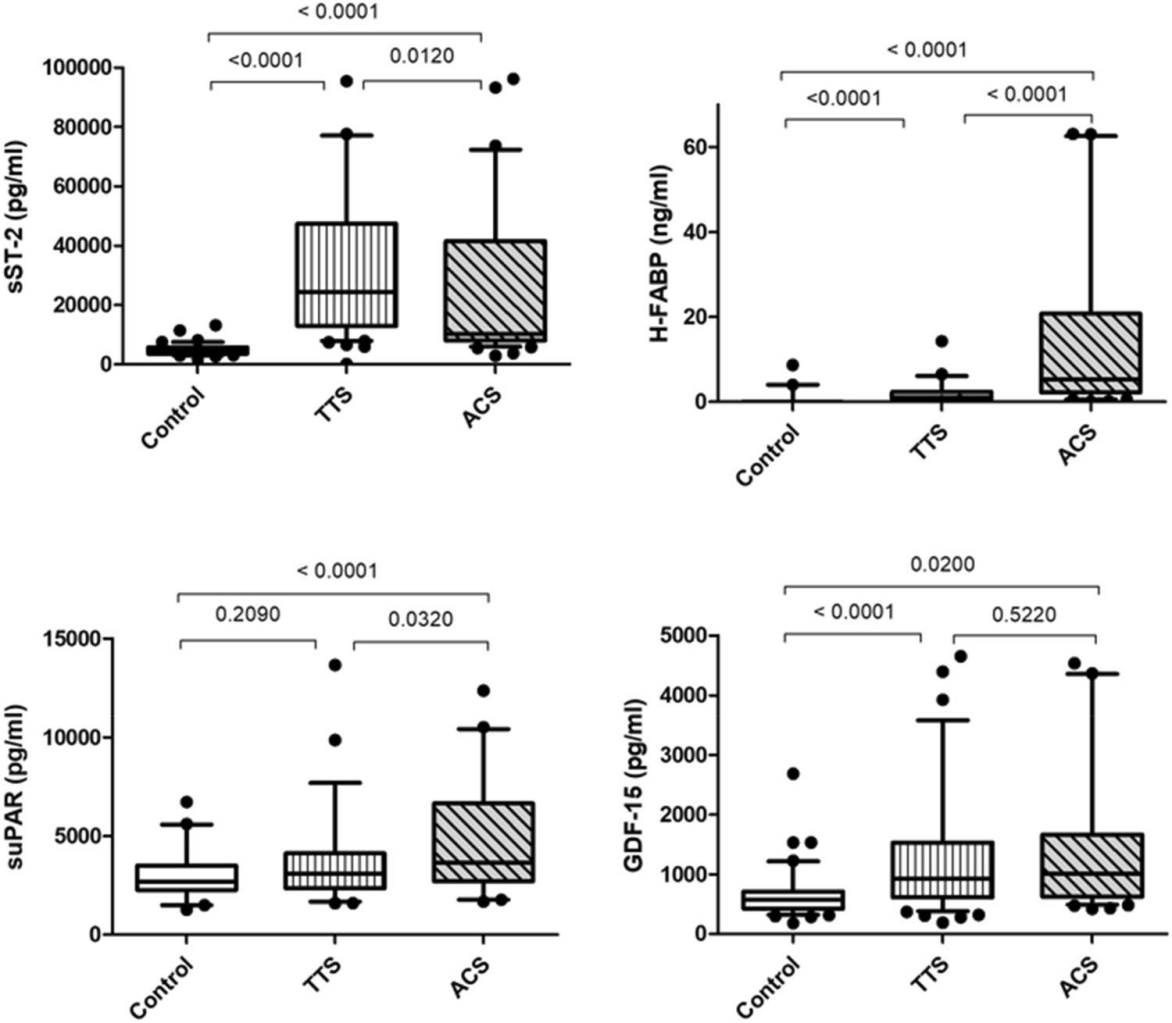
Comparison of biomarker levels between control group, TTS, and ACS patients

In contrast to suPAR, sST2, H-FABP and GDF-15, were significantly increased in patients with TTS at baseline compared to the control group (see Fig. 1 and Table 1). Whereas in the ACS group, all four markers were significantly elevated in comparison to the baseline values of controls. There was no significant difference between GDF-15 levels at admission between patients with a TTS and an ACS. H-FABP and suPAR were significantly increased in patients with an ACS compared to patients with a TTS, with the highest significance for H-FABP (*p* ≤ 0.0001). In contrast, sST-2 was significantly elevated in TTS patients compared to the ACS subgroup. In subgroup analysis of TTS and ACS patients with left ventricular ejection fraction (LVEF) of ≤ 45%, H-FABP remained significantly higher in ACS (*p* = 0.043).

### Correlations

Correlations between biomarkers and patient characteristics were shown in Table 2. Except for suPAR, a correlation of biomarkers with patient age was found. No correlation of biomarkers with BMI or creatinine levels was found. All tested biomarkers, with exception of suPAR, correlated inversely with left ventricular ejection fraction. Only GDF-15 showed a correlation with CRP levels and sST-2 an inverse correlation for HbA1c. Except H-FABP, the other biomarkers correlated inversely with LDL levels. A strong correlation was found between sST-2, suPAR, GDF-15, and H-FABP.

**Table 2:** Bivariate correlation and point-biserial correlation analysis of baseline characteristics and biomarkers

	sST-2		suPAR		GDF-15		H-FABP	
	*rs*	*p* =	*rs*	*p* =	*rs*	*p* =	*rs*	*p* =
Age (y)	1.000	0.000	0.211	0.08	0.348	0.000	0.273	0.001
BMI (kg/m^2^)	0.108	0.181	0.125	0.146	0.172	0.073	0.193	0.053
EF (%)	− 0.686	0.000	− 0.079	0.195	− 0.384	0.000	− 0.327	0.000
Creatinine (µmol/l)	0.014	0.879	0.129	0.155	0.113	0.210	0.021	0.814
CRP (mg/dl)	− 0.052	0.298	0.105	0.142	0.240	0.006	0.09	0.179
LDL (mg/dl)	− 0.302	0.000	− 0.190	0.019	− 0.152	0.049	− 0.065	0.241
HbA1c (%)	− 0.320	0.009	− 0.062	0.327	0.061	0.329	− 0.045	0.375
sST-2 (pg/ml)	1.000	0.000	0.305	0.000	0.533	0.000	0.441	0.000
GDF-15 (pg/ml)	0.533	0.000	0.383	0.000	1.000	0.000	0.516	0.000
H-FABP (ng/ml)	0.441	0.000	0.472	0.000	0.516	0.000	1.000	0.000
suPAR (pg/ml)	0.305	0.000	1.000	0.000	0.388	0.000	0.472	0.000

### ROC analysis

**Fig. 2:**
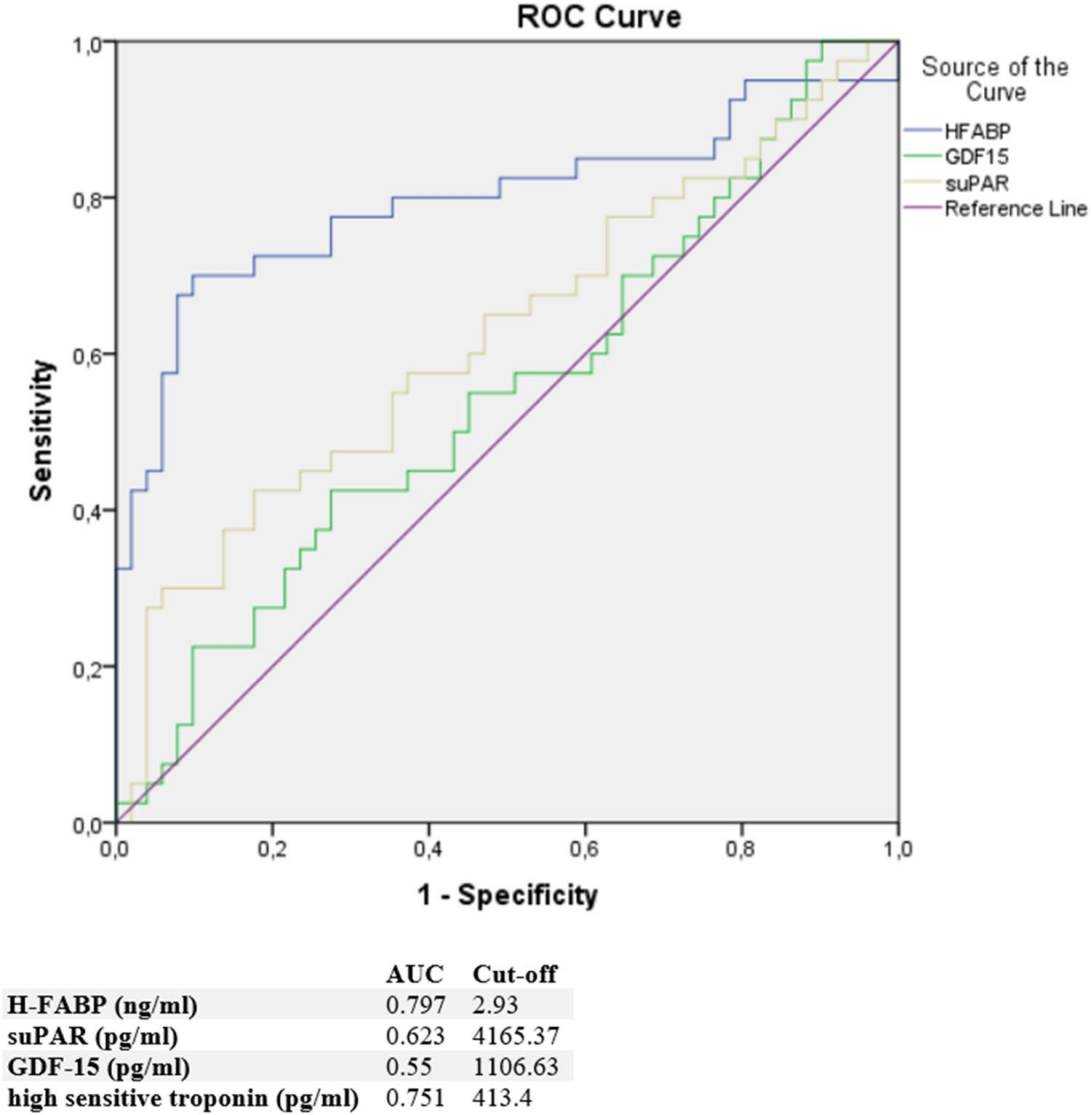
ROC curves and cut off scores for H-FABP (HFABP), suPAR, GDF-15 (GDF15) and high sensitive troponin for prediction of ACS in the total cohort

Moreover, a ROC analysis was performed and AUC was calculated for H-FABP, suPAR, GDF-15, and sST-2 levels as differential diagnostic indicators for patients presenting with chest pain in the case of either TTS or ACS. In this analysis, H-FABP was identified as the paramount biomarker for identification of an ACS when discriminating towards a TTS [AUC: 0.797 (95%Cl 0.695–0.899, *p* < 0.0001)] (see Fig. 2). An optimal cut off for diagnosis of an ACS was calculated as 2.93 ng/ml. Compared to H-FABP, suPAR evidenced a slightly lower AUC [0.623 (95% Cl 0.46–0.6, *p* = 0.046)] and GDF-15 an even lower AUC [0.55 (95% Cl 0.429–0.670, *p* = 0.419] for prediction of an ACS. In contrast, sST-2 seemed to be the most suitable biomarker [AUC: 0.653 (95% Cl 0.535–0.771, *p* = 0.012)] for prediction of a TTS in differential diagnosis to an ACS. An optimal cut off was 11,018.06 pg/ml. Rates for sensitivity, specificity, positive and negative predictive value for tested biomarkers are shown in Table 3.

**Table 3:** Rates for sensitivity, specificity, positive and negative predictive value for all tested biomarkers in ACS and TTS patients

	Sensitivity (%)	Specificity (%)	PPV (%)	NPV (%)
ACS				
H-FABP	70.0	82.4	75.7	77.4
suPAR	42.5	76.5	61.3	62.9
GDF-15	42.5	62.7	47.2	57.1
hs-Troponin	65.6	73.9	63.6	76.9
TTS				
sST-2	82.0	51.2	69.4	71.9
Pro-BNP	69.6	56.2	69.6	%

### Propensity score matching

Additionally, we performed propensity score matching for sex, left ventricular ejection fraction, and cardiovascular risk factors. Supplementary Fig. 1 depicts distribution of propensity scores between the investigated groups before and after propensity score matching, while Supplementary Fig. 2 depicts the Love plots after matching.

Notably, in the matched cohort of TTS vs. ACS, differences in the plasma levels of H-FABP and suPAR remained statistically significant (see Supplementary Table 1).

## Discussion

**Fig. 3:**
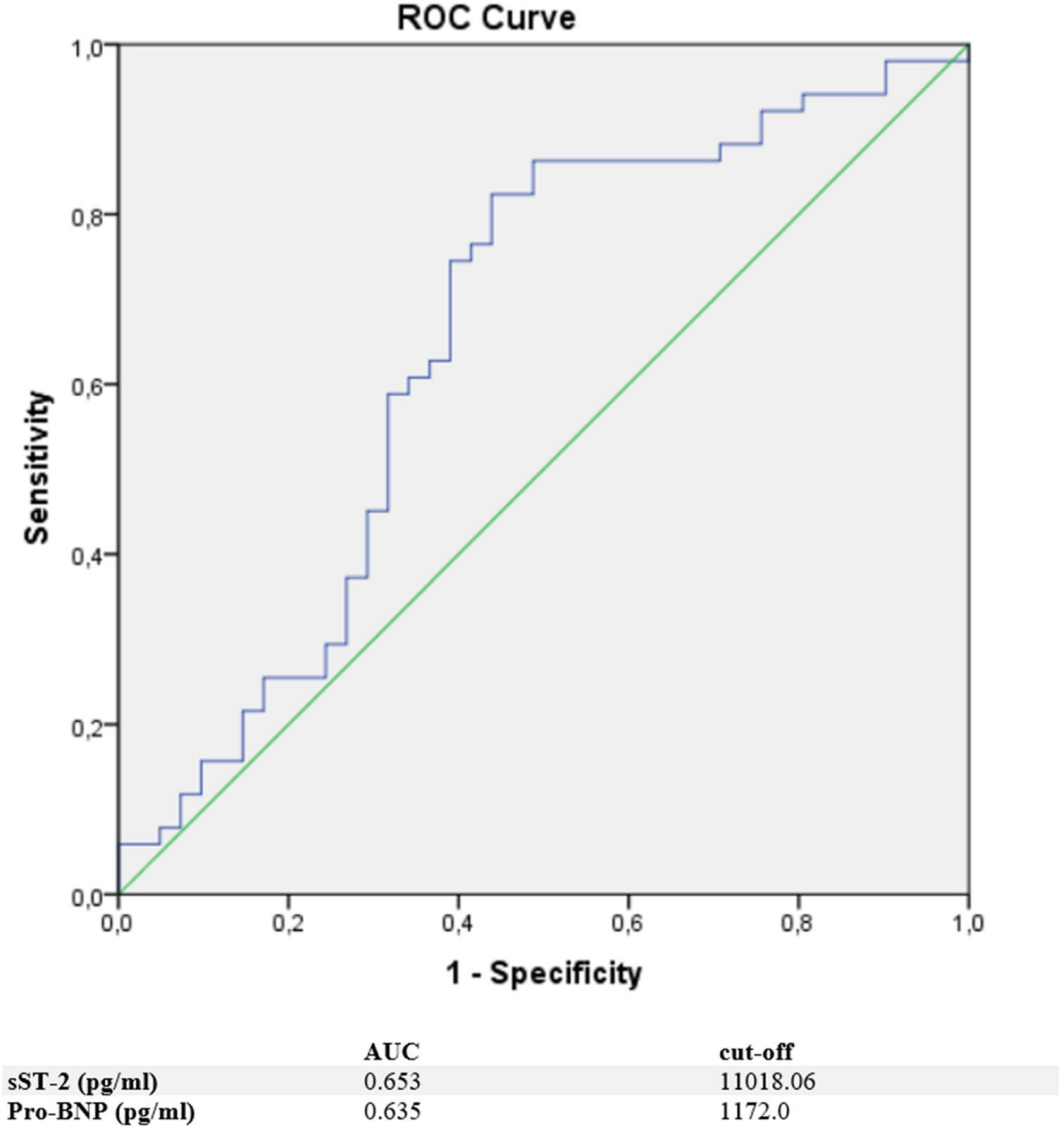
ROC curves and cut off scores for sST-2 (blue) and Pro-BNP for prediction of TTS in the total cohort

TTS is an acute heart failure condition, that resembles ACS due to its similar clinical symptoms, ECG alterations and changes in standard laboratory parameters [22]. Currently, coronary angiography is required to accurately differentiate between TTS and ACS [23]. Therefore, we aimed to investigate novel biomarkers with confirmed diagnostic value in cardiovascular diseases, which could be used in the identification of patients presenting with suspicion of TTS and effectively triage those needing urgent coronary angiography. Biomarker analysis in combination with established scoring systems, such as the Inter TAK Diagnostic Score, seems an appealing approach for more accurate triage [24]. Especially in patients with a high bleeding risk, for example, in case of neurogenic TTS due to a stroke or cerebral bleeding, or in patients with multimorbidity and advanced renal failure, avoiding coronary angiography could be of benefit (Fig. 3).

Currently available cardiovascular biomarkers are not routinely used to differentiate between TTS and ACS. The early B-type natriuretic peptide (BNP)/troponin T (TnT) ratios (specificity 95%, sensitivity 52%) and BNP/Creatinkinase-MB (CK-MB) ratios (95% specificity, sensitivity 50%) are reported to be useful in differentiating between TTS and ACS [25]. However, the ratios’ low sensitivities limit their application in clinical routine. The analysis of hs-troponin and pro-BNP in our study for differential diagnosis between TTS and ACS revealed a less favorable accuracy compared to H-FABP or sST-2 and underlines the need for novel biomarkers for differential diagnosis. Analysis of circulating microRNAs (miRNAs) has also found four miRNAs with diagnostic value in distinguishing between TTC and ACS [26]. However, despite publication in 2014, the clinical implementation of miRNAs as differential diagnostic tools has not been established in clinical routine, most likely due to the time and costs involved with the sequencing of miRNA expression profiles.

In various studies, sST-2, GDF-15, suPAR and H-FABP have been assessed as biomarkers for their diagnostic value in cardiovascular and inflammatory diseases [27]. We sought to analyze the plasma levels of sST-2, GDF-15, suPAR and H-FABP for their differential diagnostic value in patients with either ACS or TTS.

Both H-FABP and suPAR were significantly increased in patients with ACS compared to TTS, with H-FABP at a significance of *p* < 0.0001. In a subgroup analysis of TTS and ACS patients with left ventricular ejection fraction (LVEF) of ≤ 45%, H-FABP remained significantly higher in ACS (*p* = 0.043), indicating a good differential diagnostic value for patients presenting with chest pain and a high suspicion for a TTS. Furthermore, ROC analysis of H-FABP showed the highest AUC in ACS patients compared to TTS patients, making it the paramount diagnostic biomarker in our study. Optimal cut-off for H-FABP was 2.93 ng/ml (sensitivity 70.0%, specificity 82.4%). In contrast to the above-mentioned sensitivity and specificity of BNP/TnT ratios, BNP/CK-MB ratios or hs troponin, the measurement of H-FABP shows a more favorable accuracy in differentiating ACS from TTS. Furthermore, H-FABP is a highly myocardium-specific protein, which rises earlier in ACS compared to hs troponin due to its lower molecular weight. Rising H-FABP is detectable within at least 30 min following the onset of ACS, with concentrations that peak at approximately 6–8 h compared to 10–13 h for hs troponin. Besides, hs troponin’s relatively large size, the location bound within the contractile apparatus of the cardiomyocyte makes its release typically delayed for several hours after the onset of ischemic injury. This is especially of interest, when considering that myocardial markers of necrosis tend to be lower in TTS when compared to ACS and H-FABP may therefore provide better differential diagnostic value in patients presenting early with suspicion of TTS [28, 29].

Using H-FABP could avoid unnecessary coronary angiography in patients with a high bleeding risk or advanced renal failure. Furthermore, unlike troponin or pro-BNP, the lack of positive correlation of H-FABP with creatinine levels in our study makes it a valuable marker for differential diagnosis in patients with suspicion of a TTS. Together with our study results, the availability of H-FABP quick test results within 15 min, may help to establish the routine measurement of H-FABP in clinical practice, which could impact more patients that just those in whom invasive diagnostics are wished to be postponed.

The pathophysiology behind our results can be explained as H-FABP is a marker for ischemia, which is secreted by cardiomyocytes in the early phase of myocardial damage. As it indicates very early ischemic myocardial damage, H-FABP can serve as a marker for myocardial stress [30]. Baseline elevated concentrations of H-FABP indicate a strong evidence of myocardial injury as occurs in ACS [31]. In TTS, H-FABP tends to be significantly lower as transient myocardial stunning is the driving pathogenesis.

In contrast to H-FABP, sST-2 was significantly higher in TTS patients. A ROC analysis of TTS patients compared to ACS patients presented sST-2 as the most relevant diagnostic biomarker with a cut off of 11,018.06 pg/ml (sensitivity 82.0%, specificity 51.2%). Furthermore, the performance of sST2 is not influenced by renal function, as observed with Pro-BNP, and therefore provides greater diagnostic value in multimorbid patients [32]. Additionally, sST2 in heart failure is less influenced by age than Pro-BNP and may therefore be of special interest in TTS patients, as this syndrome mostly concerns older patients [33, 34]. Among the investigated biomarkers, sST-2 showed the strongest inverse correlation with the left ventricular ejection fraction, indicating that higher concentrations of sST-2 in TTS patients may reflect an exposure of mechanical stress and increased neurohormonal activation in these patients [35]. Therefore, sST-2 indicates cardiomyocyte strain and hemodynamic stress following apical, midventricular or basal akinesia in the setting of an acute TTS. Lower sST-2 plasma levels in ACS might be explained as wall motion abnormality is limited to the supply of the occluded coronary vessel in ACS patients.

These observations are in accordance with our study results, as LVEF is significantly lower in TTS compared to ACS. The lack of correlation of sST-2 with creatinine plasma levels in our study allows for its use in patients with renal insufficiency.

Thus, in particular H-FABP and sST-2 might indicate a differential diagnostic value for the guidance of treatment and therefore, further large-scale studies, investigating the value of those biomarkers are warranted. Especially, bed side tests analyzing the differential diagnostic accuracy of those novel biomarkers, may be of particular interest.

## Conclusion

Novel cardiovascular biomarkers such as H-FABP, suPAR and sST-2 offer a differential diagnostic value for distinguishing between TTS and ACS. H-FABP and sST-2 are the most promising markers with better accuracy in differential diagnosis as hs troponin/Pro-BNP in our study and, therefore, could be crucial for the guidance of treatment in patients with high bleeding risk, advanced renal failure or multimorbidity. Further evaluation of the potential clinical benefits in routine practice is necessary.

### Limitations

Major limitations of the present study are the relatively small study cohort and the fact that patients were recruited in only two study centers. Furthermore, patients of the subgroups were matched regarding gender to exclude a possible bias arising from unequal distribution. Hence, the findings of our study have to be confirmed in a “real-life population”, where ACS is more prevalent amongst male patients and large-scale studies are required to confirm the results of the present study.

## Supplementary Information

Below is the link to the electronic supplementary material.

**Suppl. Figure 1.** Propensity score distribution before/after matching (DOCX 17 KB)

**Suppl. Figure 2.** Love Plot TTS/ACS after matching (DOCX 38 KB)

**Suppl. Table 1.** Propensity score matching for sex, left ventricular ejection fraction, and cardiovascular risk factors (DOCX 53 KB)

## Data Availability

Available from corresponding author upon reasonable request.
