# Correction to: The differential diagnostic value of selected cardiovascular biomarkers in Takotsubo syndrome

**DOI:** 10.1007/s00392-021-01977-x

**Published:** 2021-12-02

**Authors:** Albert Topf, Moritz Mirna, Vera Paar, Lukas J. Motloch, Janine Grueninger, Christiane Dienhart, Paul C. Schulze, Mathias C. Brandt, Robert Larbig, Uta C. Hoppe, Daniel Kretzschmar, Michael Lichtenauer

**Affiliations:** 1 Clinic for Internal Medicine II, Department of Internal Medicine II, Paracelsus Medical University, University Hospital Salzburg, Paracelsus University Salzburg, Müllner Hauptstraße 48, 5020 Salzburg, Austria; 2 Department of Internal Medicine I, Paracelsus Medical University, 5020 Salzburg, Austria; 3 Division of Cardiology, Department of Internal Medicine I, University Hospital Jena, 07743 Jena, Germany; 4 Devision of Cardiology, Hospital Maria Hilf Moenchengladbach, 41063 Möenchengladbach, Germany

## Correction to: Clinical Research in Cardiology 10.1007/s00392-021-01956-2

In the original version of this article, the given and family names of Christiane Dienhart were interchanged. The original article has been corrected.

