# Supplementary material for: The differential diagnostic value of selected cardiovascular biomarkers in Takotsubo syndrome
Source: Clin Res Cardiol. 2021 Nov 2;111(2):197–206. doi: 10.1007/s00392-021-01956-2 (PMC8816755; doi:10.1007/s00392-021-01956-2)
Supplement: Supplementary file 1 — Suppl. Figure 1. Propensity score distribution before/after matching (DOCX 17 KB) [file 392_2021_1956_MOESM1_ESM.docx]

|  | TTS |  | ACS |  | *P*= |
| --- | --- | --- | --- | --- | --- |
|  | Median | IQR | Median | IQR |  |
| EF (%) | 45.0 | 38.8-50.0 | 43.0 | 38.0-52.0 | 0.76 |
| Age (y) | 69.0 | 58.3-76.0 | 72.0 | 62.8-79.3 | 0.622 |
| Sex (female,n) | 8/10 (80.0%) |  | 8/10  (80.0%) |  |  |
| BMI (kg/m^2) | 28.0 | 25.2-29.4 | 24.6 | 22.6-30.4 | 0.545 |
| Smoker (n) | 2/10 (20.0%) |  | 4/10 (40.0%) |  |  |
| Hypertension (n) | 9/10 (90.0%) |  | 10/10 (100.0%) |  |  |
| Diabetes (n) | 2/10 (20.0%) |  | 2/10 (20.0%) |  |  |
| sST-2 (pg/ml) | 23679.0 | 9846.6 -39023.5 | 45232.9 | 9881.7-79341.4 | 0.290 |
| H-FABP (ng/ml) | 1.1 | 0.6-2.4 | 25.8 | 10.5-60.3 | <0.001 |
| suPAR (pg/ml) | 3099.0 | 2766.9-3376.0 | 6767.4 | 4096.0-7659.3 | 0.007 |
| GDF-15 (pg/ml) | 957.3 | 529.4-1595.5 | 2532.7 | 1042.7-4405.9 | 0.023 |
