# Supplementary figures and images for: The differential diagnostic value of selected cardiovascular biomarkers in Takotsubo syndrome
Source: Clin Res Cardiol. 2021 Nov 2;111(2):197–206. doi: 10.1007/s00392-021-01956-2 (PMC8816755; doi:10.1007/s00392-021-01956-2)

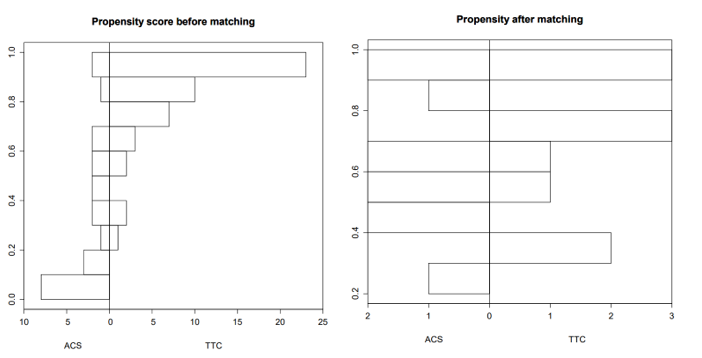

Supplement: Supplementary file 2 — Suppl. Figure 2. Love Plot TTS/ACS after matching (DOCX 38 KB) [file 392_2021_1956_MOESM2_ESM.docx]

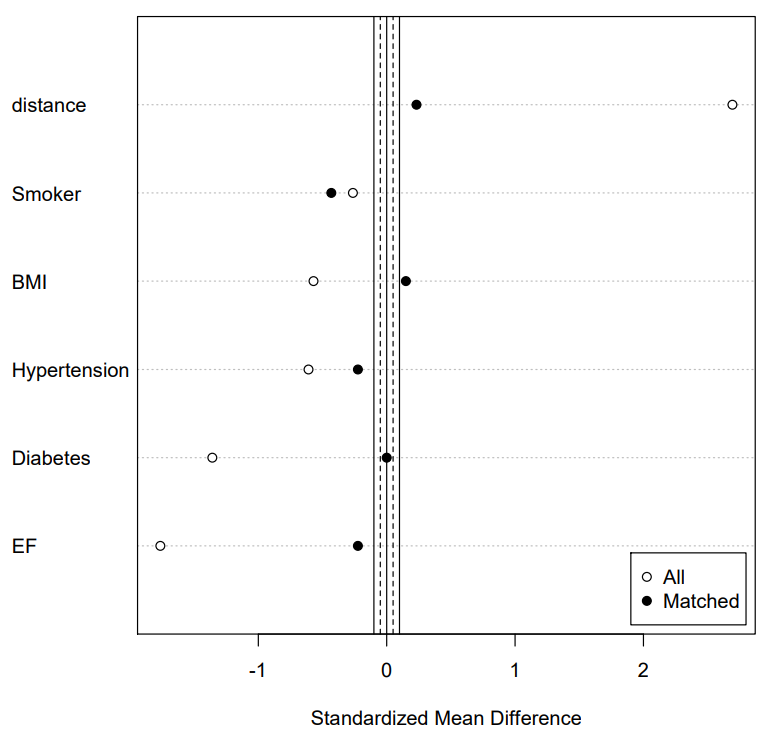

Supplement: Supplementary file 3 — Suppl. Table 1. Propensity score matching for sex, left ventricular ejection fraction, and cardiovascular risk factors (DOCX 53 KB) [file 392_2021_1956_MOESM3_ESM.docx]
